# Abnormal expression of A20 and its regulated genes in peripheral blood from patients with lymphomas

**DOI:** 10.1186/1475-2867-14-36

**Published:** 2014-04-26

**Authors:** Xu Wang, Yan Xu, Lichan Liang, Yi Xu, Chunyan Wang, Liang Wang, Shaohua Chen, Lijian Yang, Xiuli Wu, Bo Li, Gengxin Luo, Huo Tan, Wenyu Li, Yangqiu Li

**Affiliations:** 1 Key Laboratory for Regenerative Medicine of Ministry of Education, Jinan University, Guangzhou 510632, China; 2 Institute of Hematology, Jinan University, Guangzhou 510632, China; 3 Centre of Oncology and Hematology, The First Affiliated Hospital of Guangzhou Medical College, Guangzhou 510230, China; 4 Department of Oncology, First Affiliated Hospital, Jinan University, Guangzhou 510632, China; 5 Guangdong General Hospital & Guangdong Academy of Medical Sciences, Guangzhou 510080, China

**Keywords:** Lymphoma, Gene expression, MALT1, A20, NF-κB, T cell immunodeficiency

## Abstract

**Background:**

Cell-mediated immunity is often suppressed in patients with hematological malignancies. Recently, we found that low T cell receptor (TCR)-CD3 signaling was related to abnormal expression of the negative regulator of nuclear factor kappa B (NF-κB) A20 in acute myeloid leukemia. To investigate the characteristics of T cell immunodeficiency in lymphomas, we analyzed the expression features of A20 and its upstream regulating factor mucosa-associated lymphoid tissue lymphoma translocation gene 1 (MALT1) and genes downstream of NF-κB in patients with different lymphoma subtypes, including T cell non-Hodgkin lymphoma (T-NHL), B cell non-Hodgkin lymphoma (B-NHL) and NK/T cell lymphoma (NK/T-CL).

**Methods:**

Real-time PCR was used to determine the expression level of the MALT1, MALT-V1 (variant 1), A20 and NF-κB genes in peripheral blood mononuclear cells (PBMCs) from 24 cases with T-NHL, 19 cases with B-NHL and 16 cases with NK/T-CL, and 31 healthy individuals (HI) served as control.

**Results:**

Significantly lower A20 and NF-κB expression was found in patients with all three lymphoma subtypes compared with the healthy controls. Moreover, the MALT1 expression level was downregulated in all three lymphoma subtypes. A significant positive correlation between the expression level of MALT1 and A20, MALT1-V1 and A20, MALT1-V1 and NF-κB, and A20 and NF-κB was found.

**Conclusions:**

An abnormal MALT1-A20-NF-κB expression pattern was found in patients with lymphoma, which may result a lack of A20 and dysfunctional MALT1 and may be related to lower T cell activation, which is a common feature in Chinese patients with lymphoma. This finding may at least partially explain the molecular mechanism of T cell immunodeficiency in lymphomas.

## Introduction

Lymphoma is a cancer of the immune system, which includes more than 20 malignant diseases that originate from B, T or NK/T cells, and occurs via the malignant proliferation of lymphocyte clones [1]. The development, maintenance, and progression of malignant lymphomas mechanistically depend on deregulation of cellular pathways that control differentiation, proliferation, or apoptosis in lymphocytes [2]. Moreover, immune function disorders are associated with a risk for malignant transformation [1]. As previously reported, immune deficiency is one of the best characterized and strongest known risk factors for lymphoma, particularly non-Hodgkin Lymphoma (NHL). The incidence of lymphoma in people with congenital or acquired immune deficiency is 50 or more times higher than that in the healthy population [1,3]. For example, patients with weakened immune systems such as those with an HIV infection or from certain drugs or medications have a higher incidence of lymphoma [4,5]. Recently, the nuclear factor-κB (NF-κB) pathway has been considered an essential and tightly regulated signaling cascade that mediates the development, activation, and survival of normal lymphocytes for regulated immune responses [2]. Moreover, many of the oncogenic mediators involved in the pathology of lymphoma are regulated by NF-κB [6]. Abnormal NF-κB activation occurs during many pathological conditions including different abnormalities of the immune system and malignancies.

The CBM complex, including CARMA1 (caspase-recruitment domain (CARD)–containing membrane-associated guanylate kinase protein 1, also called CARD11), BCL10 (B-cell lymphoma 10) and MALT1 (paracaspase mucosa-associated lymphoid tissue lymphoma translocation gene 1), is crucial for TCR–induced NF-κB and T cell activation [7-10]. The CBM pathway is pathologically altered in several lymphoma subtypes, such as activated B-cell-like diffuse large B-cell lymphoma (ABC-DLBCL). The CBM complex mediates activation of the inhibitor of NF-κB (IκB) kinase complex (IKK) by ubiquitylation and phosphorylation events that depend on TRAF6 and TAK1, respectively. The IKK-mediated phosphorylation of IκBα targets the inhibitor for proteasomal degradation and allows the nuclear translocation of NF-κB [10,11]. Studies in MALT1-deficient mice have demonstrated an essential role for MALT1 in TCR- and BCR-mediated functions [10,11].

The intracellular ubiquitin-editing protein A20 (also known as tumor necrosis factor alpha-induced protein 3, TNFAIP3) is a key player in the negative feedback regulation of NF-κB signaling [12-14]. A20 was identified as a MALT1 substrate, and MALT1 can cleave A20 at arginine 439 to impair its NF-κB inhibitory function, thus emphasizing the importance of the MALT1 proteolytic activity in the ‘fine tuning’ of T cell antigen receptor signaling [7,11]. Subsequent studies demonstrated that A20 overexpression inhibits NF-κB activation in response to different stimuli [15,16]. The cloning and characterization of the A20 promoter revealed the presence of two NF-κB DNA binding elements, which are recognition sequences for NF-κB transcription factors. It was also found that multiple NF-κB activating stimuli induce A20 expression via NF-κB sites in the A20 promoter [17]. Additionally, recent genome-wide association studies have demonstrated a strong link between A20 polymorphisms and a range of chronic inflammatory disorders including autoimmune diseases, such as systemic lupus erythematosus (SLE) and rheumatoid arthritis (RA) [14]. SLE and RA are associated with a significantly increased risk for lymphoma, particularly MALT lymphoma [13,14]. Moreover, A20 dysfunction by deletion or mutation has been identified in numerous lymphocytic malignancies. The A20 mutations identified in lymphoma, which are distributed throughout the gene, affect the ovarian tumour (OTU), ZnF and linker regions in DLBCL, mantle cell lymphoma (MCL), mucosa-associated lymphoid tissue (MALT), classic Hodgkin’s lymphoma (cHL), marginal zone lymphoma (MZL) and primary mediastinal B cell lymphoma (PMBL) [5].

The etiology of lymphoma remains to be understood; however, both aberrant NF-κB activation and a weakened immune system can promote the malignant transformation of lymphocytes. In contrast, cell-mediated immunity is often suppressed in patients with lymphomas and may be related to disease progression. Such immune dysfunction may be due to disorders in thymic output function and T cell proliferation and activation [18-20], and their molecular mechanism remains unclear. Few studies have focused on the expression characteristics of A20, its regulatory factor MALT1 and NF-κB in patients with lymphomas and evaluated immune function in patients. In this study, we analyzed the expression level of all three genes in samples from Chinese patients with T cell NHL (T-NHL), B cell NHL (B-NHL), and NK/T cell lymphoma (NK/T-CL) to further understand the role of A20 and the NF-κB pathway in the occurrence and development of lymphoma.

## Methods

### Samples

Fifty-nine cases with lymphomas (37 males and 22 females with a median age of 48 years and a range of 12–78 years), including T-NHL (24 cases), B-NHL (19 cases) and NK/T-CL (16 cases), were selected for this study. Thirty-one healthy individuals (HI) served as control. Characteristics of lymphoma samples and healthy control were summarized in Table 1. All of the procedures in this study were conducted according to the guidelines of the Medical Ethics committee of the Health Bureau of Guangdong province, China.

**Table 1:** Characteristics of lymphoma samples and healthy control

**Diagnosis**	**n**	**Gender**		**Age (year)**		**Stage**			
		**Male**	**Female**	**Median**	**Range**	**I**	**II**	**III**	**IV**
**T-NHL**	24	12	12	42	12-78	9	1	1	13
**B-NHL**	19	12	7	56	21-78	3	3	5	8
**NK/T-CL**	16	13	3	39	13-74	3	2	1	10
**HL**	31	17	14	38	25-72				

Note: *T-NHL* T cell non-Hodgkin lymphoma, *B-NHL* B cell non-Hodgkin lymphoma, *NK/T-CL* NK/T cell lymphoma, *HI* healthy individuals

Peripheral blood samples were collected by heparin anticoagulation, and peripheral blood mononuclear cells (PBMCs) were isolated using the Ficoll–Hypaque gradient centrifugation method. The percentage of CD3 + cells in PBMCs was found around 70%. RNA extraction and cDNA synthesis were performed according to the manufacturer’s instructions.

### Quantitative real-time RT-PCR (qRT-PCR)

The sequences of primers used for MALT1, A20 and NF-κB gene amplification are listed in Table 2. There are two variants of the MALT1 gene, MALT1-V1 and MALT1-V2, and the latter contains a 33 bp deletion located between exons 6 and 8. To amplify the two MALT1 transcript variants, the MALT-V1-for and MALT-V1-rev primer pair was designed for MALT1-V1 amplification to amplify the region that is missing in MALT1-V2, and the MALT1-for and MALT1-rev primer pair was designed to amplify the conserved region, which is found in both variants [21].

**Table 2:** List of primers

**Primer**	**Sequence**	**Accession no.**	**PCR productsize**
A20 For	5′-CTGGGACCATGGCACAACTC-3′	NM_006290	182 bp
A20 Rev	5′-CGGAAGGTTCCATGGGATTC-3′		
MALT1-V1 For	5′-AAGCCCTATTCCTCACTACCAG-3′	NM_006785.2	195 bp
MALT1-V1 Rev	5′-CACTCCACTGCCTCATCTGTTC-3′		
MALT1 For	5′-TCTTGGCTGGACAGTTTGTGA-3′	NM_006785.2	230 bp
MALT1 Rev	5′-GCTCTCTGGGATGTCGCAA-3′		
NF-κB For	5′-CCACAAGACAGAAGCTGAAG-3′	NM_003998	149 bp
NF-κB Rev	5′-AGATACTATCTGTAAGTGAACC-3′		
β2M For	5′-TACACTGAATTCACCCCCAC-3′	J00105	145 bp
β2M Rev	5′-CATCCAATCCAAATGCGGCA-3′		

The expression level of the A20, MALT1, MALT1-V1, NF-κB and β2-microglobulin (β2M) genes was determined by SYBR Green I real-time PCR. Briefly, PCR was performed in a 20 μl volume with approximately 1 μl of cDNA, 0.5 μM of each primer pair, 9 μl of 2.5 × Real Master Mix (Tiangen Biotech (Beijing) Co. Ltd., Beijing, China) and 9 μl of dH_2_O. After initial denaturation at 95°C for 15 minutes, 45 cycles of the following procedure was performed: 30 seconds at 95°C and 40 seconds at 60°C. The plate was read immediately after the 60°C step using an MJ Research DNA Engine Opticon 2 PCR cycler (Bio-Rad, Hercules, CA, USA). The relative amount of the genes of interest and β2M reference gene was measured in two independent assays. The specific, amplified PCR products were analyzed by melting curve analysis. The data are presented as the relative expression of the genes of interest compared with the internal control gene as determined by the 2(^-△CT^) method [22]. In addition, to analyze the MALT1-V1 expression characteristics, we calculated the MALT1-V1 expression ratio as MALT1-V1/MALT1 x 100%.

### Statistical analysis

Two independent-samples Wilcoxon tests were performed to compare the median expression level for each gene between patients with T-NHL, B-NHL and NK/T-CL and the control group. Spearman correlation and linear regression analyses were used to determine the association between different genes in different groups. A P <0.05 was considered statistically significant [20].

## Results and discussions

**Figure 1:**
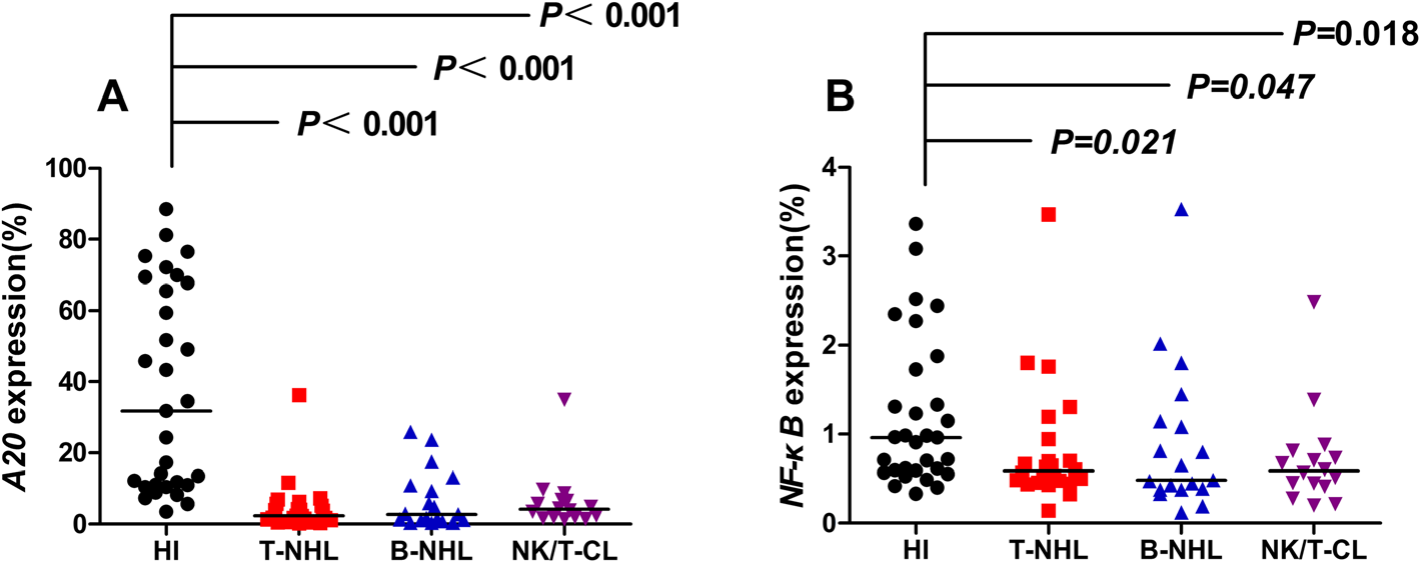
The A20 (A) and NF-κB (B) expression level in patients with T-NHL, B-NHL and NK/T lymphoma and healthy individuals.

Like many cancers and leukemias, patients with lymphoma have cell-mediated immune dysfunction [23]. Such T cell immunodeficiency may at least be partially related to low T cell receptor (TCR)-CD3 signaling [24,25]. Recently, we found abnormal expression of MALT1, A20, and NF-κB genes, which may be related to T cell immunodeficiency, in T cells from patients with acute myeloid leukemia (AML) [21]. In this study, we analyzed the feature of gene expression pattern in PBMCs from lymphoma samples, unlike leukemia samples, in which the most cells in PBMCs are leukemia cells, in this case, T cells must be sorted from PBMCs [21], while the high percentage of CD3 + T cells in PBMCs is thought that could be represented the characteristics of T cells specially discussing the T cell related pathways. In this study, we analyzed the expression of A20 in 59 patients with different lymphoma subtypes (T-NHL, B-NHL and NK/T-CL), and a significantly lower A20 expression level (median: 2.967) was found in all lymphoma samples compared with those in healthy individuals (median: 31.754, *P <* 0.001). Moreover, there was also a significantly lower A20 expression level in each T-NHL, B-NHL and NK/T-CL sample (median: 2.403, 2.816, and 4.183, respectively) compared with those from the healthy group (*P <* 0.001 for all comparisons) (Figure 1A). These results are similar to a previous finding in AML [21]. It was suggested that the decreased A20 in AML may be due to activation of a subset of T cells, which is thought to be a specific response to AML cells, and the presence of clonally expanded T cells in AML and other leukemias may support this hypothesis [21,26-29]. In this study, the lower A20 expression may be similar to findings in T cells from AML patients related to lower lymphocyte activation, and our previous finding demonstrating TCR subfamily T cell proliferation may also support these results [18]. Moreover, A20-deficient mice have high oncogenic risk [6,12]. Further follow up of the association between the expression characteristics of A20 and disease progression is needed.

It is well known that NF-κB overexpression plays a key role in the development of lymphocytic malignant cells [3,23] and lymphocytes, which mediate inflammation [30]. However, little is known about the expression characteristics of NF-κB in normal lymphocytes from patients with lymphocytic malignancies who have T cell immunodeficiency. Interestingly, in this study, we found that the NF-κB expression level in PBMC samples from patients with T-NHL, B-NHL and NK/T-CL (median: 0.584, 0.484, and 0.584, respectively) was decreased. Significantly lower NF-κB expression was found in patients with T-NHL (*P* = 0.021), B-NHL (*P* = 0.047) and NK/T-CL (*P* = 0.018) in comparison with healthy individuals (median: 0.962) (Figure 1B). This result may further suggest lower lymphocyte activation in lymphoma patients, and it appears that lower lymphocyte activation may be in all of three type lymphomas. However, this result is inconsistent with the finding of lower A20 expression in samples from the same lymphoma patients. Because A20 inhibits NF-κB, lower expression of this protein may reduce its NF-κB inhibitory effects, and NF-κB may be upregulated [2,31]. This phenomenon may exist for two different reasons. First, there is significantly lower lymphocyte activation, particularly in T cells in lymphoma patients, even when A20 is downregulated, and these cells may be incapable of upregulating the expression of NF-κB; second, it may be due to the abnormal expression of a different NF-κB regulator because NF-κB is affected by numerous regulatory factors e.g., MALT1 [6,11]. Moreover, MALT1 is an upstream A20 pathway factor that cleaves A20 at arginine 439 and impairs its NF-κB inhibitory function [32].

**Figure 2:**
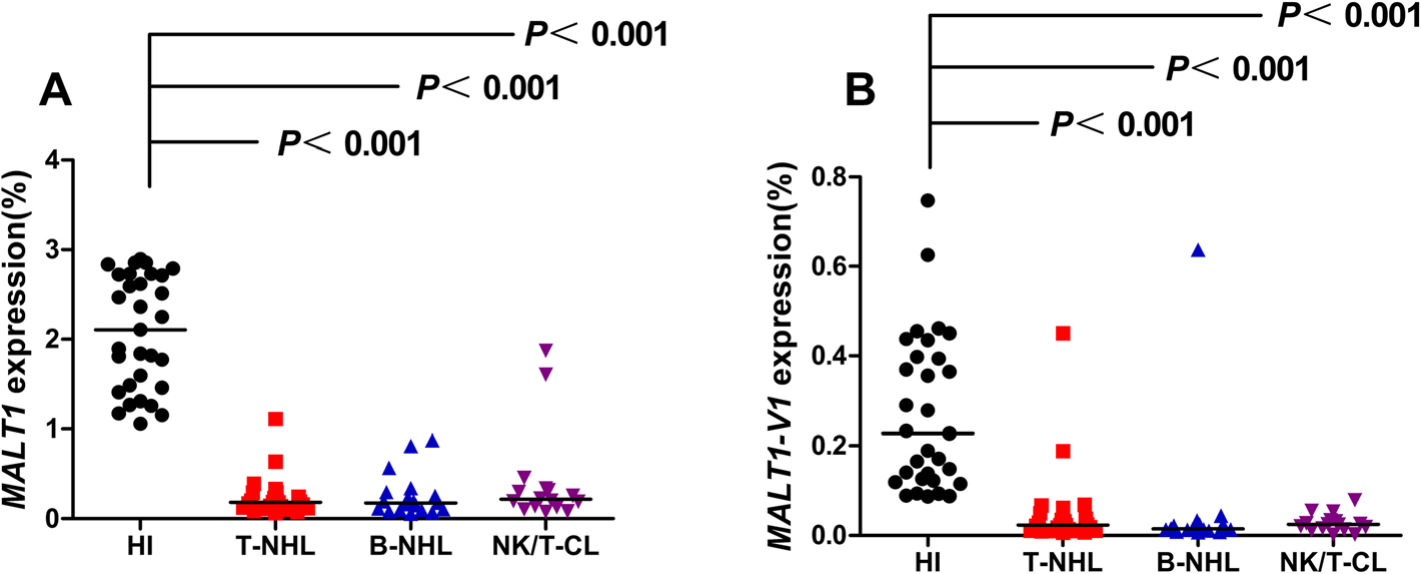
The expression level of MALT1 (A) and MALT1-V1 (B) in patients with T-NHL, B-NHL and NK/T lymphoma and healthy individuals.

To characterize the relationship between MALT1, A20 and NF-κB, we examined the MALT1 expression level. As expected, the MALT1 expression level was significantly downregulated in patients with T-NHL (median: 0.186) (*P <* 0.001), B-NHL (median: 0.177) (*P <* 0.001) and NK/T-CL (median: 0.217) (*P <* 0.001) compared with healthy individuals (median: 2.105) (Figure 2A). Because MALT1 is a positive regulatory factor of NF-κB, its lower protein expression may result in the downregulation of NF-κB, which is further supported by the finding of a lower NF-κB level in patients with lymphoma. However, this result appears to be inconsistent with the lower expression level of A20, which was identified as a MALT1 substrate and could be cleaved by MALT1 [7]. This finding is similar to phenomena in T cells in AML patients, which may indicate that there is more than one A20 regulator. For example, there are two MALT1 variants, MALT1-V1 and MATL1-V2, and little is known about the functional difference between the variants. Our previous study found that the MALT1-V1 expression level was significantly higher in T cells from AML patients compared with healthy controls, while the MALT1-V2 expression level was downregulated [21]. In this study, we also analyzed the expression level of the MALT1 variants. In contrast with the finding in T cells from AML patients, a significantly lower MALT1-V1 expression level was detected in patients with T-NHL, B-NHL and NK/T-CL (median: 0.023, 0.015, and 0.024, respectively) compared with healthy controls (median: 0.227, *P <* 0.001 for all comparisons) (Figure 2B). Because we could not directly amplify MALT1-V2, which has a 33 bp deletion, the MALT1-V2 expression level could only be indirectly calculated by the relative expression of MALT1-V1/total MALT1 [21], and there was no significant difference in the MALT1-V1/total MALT1 ratio between patients with T-NHL, B-NHL and NK/T-CL and healthy controls (median: 12.46, 11.54, and 8.87%, respectively, vs. 12.63%), implying that the MALT1-V2 expression level was also downregulated in T-NHL, B-NHL and NK/T-CL. There are no previous reports describing the expression pattern, distribution, or different biological functions of the MALT1 variants in the literature before our first report that described the change in the expression pattern of MALT1 variants in T cells from patients with AML [21]. In this study, we found that the expression ration of MALT1-V1 in total MALT1 was14.32 ± 13.21% (median: 11.54%) in lymphoma group, while 12.95 ± 7.10% (median: 12.63%) in healthy group, indicating the common feature that expression levels of MALT1-V1 was low than MALT1-V2 in PBMCs from all samples. The different biological functions of both MALT1 variants is needed further investigation.

**Figure 3:**
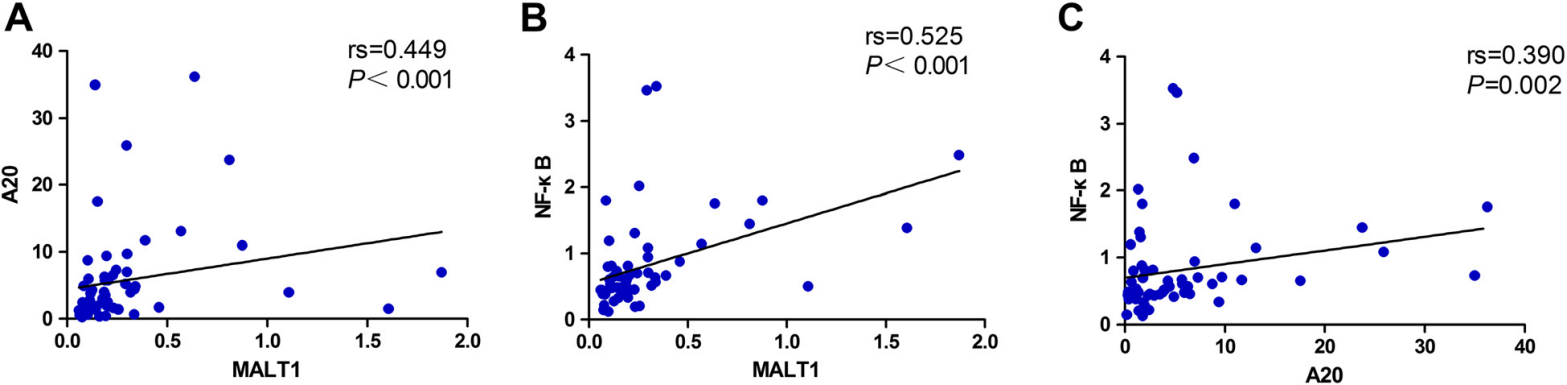
Correlation between the gene expression levels of MALT1 and A20 (A), MALT1 and NF-κB (B) and A20 and NF-κB (C) in patients with T-NHL, B-NHL and NK/T lymphoma.

Overall, either MALI1-V1 or MALT1-V2 was decreased in lymphomas. Thus, our finding of lower MALT1 expression may imply a loss of control of T cell activation and even progression toward immune deficiency in lymphoma patients. We further analyzed the correlation between the MALT1 and A20 expression level. In general, A20 is cleaved by MALT1; thus, the expression level of MALI1-V1 and MALT1-V2 should be negatively correlated with the A20 and MALT1 expression pattern [32]. However, we found a positive correlation between MALT1 and A20 (rs = 0.449, *P <* 0.001) (Figure 3A) and MALT1-V1 and A20 (rs = 0.295, *P* = 0.023) in 60 lymphoma patients. This result implied the abnormal regulation of MALT1 and A20. Moreover, a positive correlation was found between MALT1 and NF-κB (rs = 0.525, *P <* 0.001) (Figure 3B) and A20 and NF-κB (rs = 0.390, *P* = 0.002) (Figure 3C), indicating that MALT1, A20, and NF-κB lost their normal expression pattern at the molecular level and may be more complex in their manner of regulation in lymphoma. Further investigation is needed to characterize the upstream pathway regulators of A20 in addition to MALT1.

In conclusion, we characterized, for the first time, the alternative expression pattern of MALT1, A20 and NF-κB, which may be related to abnormal T cell activation in lymphomas. A lack of A20 and dysfunctional MALT1, which results in lower T cell activation, are common characteristics in Chinese patients with T-NHL, B-NHL and NK/T-CL, and this combination may at least partially explain the molecular mechanisms involved in T cell immunodeficiency in lymphomas. These findings may help provide new data to consider for target immune regulation in lymphoma. However, further investigation is needed to follow up on patients with different MALT1-A20-NF-κB expression patterns and their association with cancer development.

## Competing interests

The authors declare that they have no competing interests.

## Authors’ contributions

YQL contributed to the concept development and study design. XW, YX, LCL and YX performed real-time PCR, SHC and LJY prepared PBMCs, XLW and BL prepared RNA and cDNA, CYW, LW, HT, GXL and WYL was responsible for clinical diagnoses and performed clinical data acquisition. XW, WYL and YQL coordinated the study and helped draft the manuscript. All authors read and approved the final manuscript.
